# Effect of Computer-Assisted Cognitive Remediation Therapy on Cognition among Patients with Schizophrenia: A Pilot Randomized Controlled Trial

**DOI:** 10.3390/biomedicines12071498

**Published:** 2024-07-05

**Authors:** Ayumi Yamanushi, Takeshi Shimada, Ami Koizumi, Masayoshi Kobayashi

**Affiliations:** 1 Medical Corporation Seitaikai, Mental Support Soyokaze Hospital, Nagano 386-0401, Japan; a_a_a_yumi63@yahoo.co.jp (A.Y.); shimadatakeshi0703@yahoo.co.jp (T.S.); 7amy1128@gmail.com (A.K.); 2 Department of Health Sciences, Graduate School of Medicine, Shinshu University, Nagano 390-8621, Japan

**Keywords:** cognition, cognitive remediation, randomized controlled trial, rehabilitation, schizophrenia

## Abstract

In schizophrenia, cognition is closely linked to social competence and influences long-term prognosis. Thus, treatment should target cognitive improvement to enhance the patient’s societal adaptation. This study evaluated the effects of computer-assisted cognitive remediation therapy (CR) using RehaCom^®^ on cognition in patients with schizophrenia. Thirty patients were randomized, with 15 assigned to the CR and treatment as usual (TAU) group and 15 to the TAU-alone group. Over 12 weeks, patients received CR twice weekly, including two computer sessions and one verbal session. The outcomes measured were cognition using the Brief Assessment of Cognition in Schizophrenia and Schizophrenia Cognition Rating Scale, intrinsic motivation using the Quality of Life Scale and Intrinsic Motivation Inventory, psychiatric symptoms using the Positive and Negative Syndrome Scale, negative symptoms using the Scale for the Assessment of Negative Symptoms, and functional level using the modified Global Assessment of Functioning scale for Functioning. The CR + TAU group demonstrated considerable improvements in cognition, intrinsic motivation, and functional level compared to the TAU-alone group. These findings indicate that the CR using RehaCom^®^ enhances cognition and other outcomes in schizophrenia.

## 1. Introduction

Cognitive impairment is a central feature of schizophrenia, significantly impacting functional outcomes [1,2,3]. Most patients experience deficits in attention/vigilance, verbal learning and memory, executive functioning, verbal fluency, and processing speed [4,5]. The severity of these impairments is consistently linked to various functional aspects and long-term disability [6,7,8]. Treatment has primarily focused on pharmacological and non-pharmacological approaches. Psychosocial treatments for cognitive remediation (CR) have shown more promising results compared to pharmacological methods [9,10,11,12,13,14].

CR therapy methodologies vary and include bottom-up vs. top-down, computer-based vs. non-computer-based, group vs. individual, tailored vs. standardized, and combined vs. not combined with other treatments [14,15,16,17]. A meta-analysis by Twamley et al. [18] showed that the effect size of computer-assisted CR therapy on cognition was greater than that of non-computer-assisted therapy. While the benefits of CR therapy are recognized, more information is required on effective CR therapy methodologies and related factors to formulate evidence-based recommendations for enhancing cognition in patients with schizophrenia.

We devised a CR therapy program utilizing the Japanese version of the Higher Brain Function Training System RehaCom^®^ “http://www.hasomed.de (accessed on 20 December 2019)”. While RehaCom^®^-based CR therapy has been used for patients with schizophrenia in other countries [19,20,21], the Japanese version was introduced in September 2019, and its impact on patients with schizophrenia in Japan remains unexplored. This study sought to assess the feasibility and effectiveness of augmenting treatment as usual (TAU) with RehaCom^®^-based CR therapy compared to TAU alone for cognition in patients with schizophrenia.

## 2. Materials and Methods

### 2.1. Study Design and Procedures

This randomized, parallel two-arm, single-blind, controlled trial evaluated the effect of adding RehaCom^®^-based CR therapy to TAU on cognition and other outcomes compared to TAU alone in patients with schizophrenia. This study was conducted between December 2021 and July 2022 at the Medical Corporation Seitaikai Mental Support Soyokaze Hospital in the Nagano Prefecture, Japan, a suburban community.

Prospective patients met with the study team, reviewed the study procedures, and if interested, gave informed consent and scheduled a baseline assessment. After completing the baseline assessment, eligible patients were randomized into the CR + TAU and TAU-alone groups. Post-treatment assessment occurred 12 weeks after baseline.

### 2.2. Participants

Between January 2021 and June 2022, participants were recruited from inpatients and outpatients at the Medical Corporation Seitaikai Mental Support Soyokaze Hospital. The inclusion criteria were patients 20–60 years of age and diagnosed with schizophrenia based on the Structured Clinical Interview for DSM-5 Disorders Research Version (SCID-5-RV) [22,23]. The exclusion criteria were patients with current primary DSM-5 diagnoses other than schizophrenia, mental retardation, a history of neurological disorders, active substance abuse within six months before consent, a history of psychosis accounted for by substance abuse, a current risk of suicide, and comorbid serious physical disorders.

### 2.3. Randomization and Blinding

Eligible participants were randomized into the CR + TAU and TAU-alone groups by independent study staff, with whom there was no patient contact, using a computer-generated randomization program. Randomization was stratified by sex (male/female) and age (20–29, 30–39, 40–49, 50–59, and 60–65 years). Within each stratum, participants were randomized 1:1 to TAU + AE or TAU alone. The treatments were open-label; however, the assessors were blinded to the treatment allocation.

### 2.4. Interventions

#### 2.4.1. CR Therapy

The CR therapy included computer and verbal sessions. The modules of the computer-assisted RehaCom^®^-based CR therapy modules included 24 individual 60 min sessions, 2 sessions/week, over 12 weeks. CR therapy was conducted on a computer with a special input panel using the Japanese version of the RehaCom^®^ software package Ver.1.0 (KISSEI COMTEC Co., Ltd., Matsumoto, Japan). RehaCom^®^ is a computer system for continuous cognitive training such as attention, memory, executive function, and visual perception. Each RehaCom^®^ module was classified according to the categories established by the MATRICS^TM^ Consensus Cognitive Battery (MCCB^TM^) [24,25]: speed of processing, attention/vigilance, working memory, verbal learning and memory, visual learning and memory, reasoning and problem-solving, and social cognition (see Appendix A, Table A1). The characteristics of RehaCom^®^ include a highly game-like training menu and a function that automatically adjusts the difficulty level according to the patient’s performance so that patients always receive the appropriate stimulation and continue to work on the training modules with motivation. Occupational therapists certified as cognitive remediation specialists administered the CR therapy. Patients used a desktop computer and an ergonomic keyboard. Each training session was divided into modules selected by the therapists in various cognitive domains. Appendix B, Table A2 provides the details of the treatment schedule for CR therapy. Further details of the RehaCom^®^ software are described at http://www.hasomed.de (accessed on 20 December 2019). In addition to the computer sessions using RehaCom^®^, one-on-one verbal sessions to bridge the gap between improvements in cognitive impairment and daily functioning [12,13,26] were conducted with the therapist. Bridging interventions aid patients in applying their cognition to their daily functioning and promote socialization. The topics in this intervention included community living, the role of cognition in social skills, and problem-solving concerning compensatory strategies for dealing with daily living challenges. CR therapy was considered complete when patients participated in a minimum of 80% of the 24 scheduled treatment sessions.

#### 2.4.2. TAU

All patients received standard treatment throughout the study, including regular meetings with a psychiatrist, antipsychotic medication, individual case management, and rehabilitation programs such as occupational therapy, at the same frequency as the CR + TAU group. Patients in the TAU-alone group received the same number of hours and amount of TAU intervention as those in the TAU + CR group.

### 2.5. Outcomes

#### 2.5.1. Feasibility and Acceptability Outcomes

The feasibility and acceptability outcomes were the number of eligible patients randomized among the referred and recruited patients, retention rate by the proportion of participants with outcome measures at post-intervention and data completion, acceptability of treatments by dropout from the allocated group, and treatment adherence rate by the proportion of participants receiving a threshold dose of the CR therapy intervention (80% or more) in the CR + TAU group. CR therapy implemented in >80% of the patients indicated good adherence to CR therapy protocols.

#### 2.5.2. Efficacy Outcomes

Efficacy outcomes were collected at baseline and 12 weeks. The primary efficacy outcome was the change in cognition examined from the baseline to 12 weeks using the Brief Assessment of Cognition in Schizophrenia (BACS). The secondary efficacy outcomes included changes in intrinsic motivation, psychopathology, and functional levels from baseline to 12 weeks.

Cognition was examined with the BACS [27,28] and the Schizophrenia Cognition Rating Scale (SCoRS) [29,30,31]. The BACS consists of six domains: verbal memory (list learning), working memory (digit sequencing task), motor speed (token motor task), verbal fluency (category instances and letter fluency), attention and processing speed (symbol coding), and executive function (Tower of London test). The Z-scores were created to standardize each of the six domains, wherein the mean of healthy controls was set to zero and the standard deviation was set to one. The composite score was calculated as the average Z-score for each of the six BACS measures. The SCoRS is a 20-item interview-based measure of cognitive impairment with questions aimed at the degree to which this impairment impacts daily functioning. Additionally, a global rating, which reflects the overall impression of the level of cognitive difficulty of patients in 20 cognition areas, was generated. The items were developed to examine the cognitive domains of memory, learning, attention, working memory, problem-solving, motor skills, social cognition, and language production. Each item was rated on a scale ranging from 1 to 4, and the global rating ranged from 1 to 10, with higher ratings reflecting greater impairment. The anchor points for each item focused on the impairment degree in that ability and the degree to which the deficit impaired daily functioning. The evaluators only considered cognitive deficits and attempted to rule out non-cognitive sources of deficits. The complete SCoRS administration included three various ratings: ratings based on an interview with the patient, an interview with an informant (i.e., the person who had the most regular contact with the patient in everyday situations), and generated by the interviewer who administered the scale to the patient and informant. The SCoRS total is the sum of the 20 SCoRS items.

Intrinsic motivation was examined using the sum of the following three items from the Quality of Life Scale (QLS), sense of purpose, motivation, and curiosity [32,33,34], as well as by using the Intrinsic Motivation Inventory (IMI) [35]. The QLS is rated on a scale ranging from 0 to 6, with higher scores indicating better functioning. The IMI is a 21-item self-reporting scale that measures interest/enjoyment, value/usefulness, and perceived choice. The items were answered on a 7-point Likert scale with responses ranging from “not at all true” to “very true” with a higher total score reflecting greater intrinsic motivation for a specified task.

Psychiatric symptoms were examined using the Positive and Negative Syndrome Scale (PANSS) [36], which is a 30-item rating scale designed to assess psychotic symptom severity and consisting of three domains: positive, negative, and general psychopathology. Each item is rated from 1 to 7, with higher scores indicating more severe symptoms.

Negative symptoms were assessed with the Scale for the Assessment of Negative Symptoms (SANS) [37], which consists of 30 items that result in global ratings in five symptom complexes: affective flattening, alogia, avolition/apathy, anhedonia/asociality, and attention. Each item and its global ratings range from 0 to 5, with higher scores indicating more severe negative symptoms.

The functional levels were examined using the modified Global Assessment of Functioning (mGAF-original) scale [38]. Eguchi et al. [39] translated the original mGAF [38] into Japanese and developed the psychological symptom (mGAF-S) and social functioning (mGAF-F) subscales by splitting the items and anchor points of the original mGAF scale. We used the mGAF-F, a single-item rating scale, to measure the functioning of the patients. Scores on each scale are rated between 21 and 90, with higher scores indicating better functioning.

### 2.6. Statistical Analysis

We summarized the descriptive statistics for the feasibility outcomes. T-tests for continuous data and χ^2^ tests for categorical data were used to determine the group differences in sociodemographic and clinical characteristics. We performed an analysis of covariance (ANCOVA) with an intention-to-treat analysis of each outcome measure using all available data to evaluate the efficacy outcomes. We conducted an ANCOVA with the post-intervention assessment score as the dependent variable and the baseline score, age, sex, baseline IMI total, and baseline QLS total as covariates to examine group differences in the primary efficacy outcome. Moreover, we examined group differences in secondary efficacy outcomes using ANCOVA, with post-intervention scores as the dependent variable and baseline scores, age, and sex as the covariates. Treatment effects were estimated at the 12-week point, with an interaction between group and time. The Bonferroni method was used for multiple comparisons. Furthermore, we calculated the effect sizes with ηp^2^ for intervention changes. Statistical significance was set at *p* < 0.05 for a two-sided test. Statistical analyses were performed using SPSS Statistics version 28.0. (IBM, Armonk, NY, USA).

## 3. Results

### 3.1. Participants

**Figure 1:**
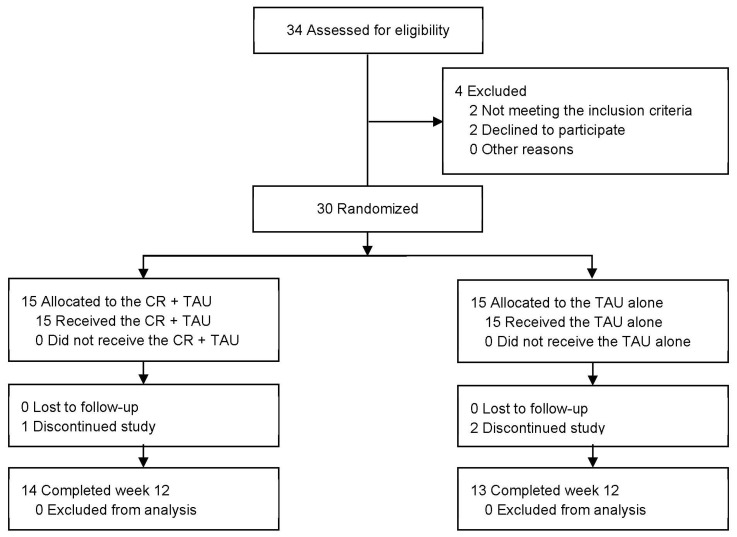
Study flowchart. CR, cognitive remediation; TAU, treatment as usual.

Figure 1 illustrates a flowchart of the study. Of the 34 patients enrolled in the trial, 30 met the inclusion criteria. Among the 30 patients who were randomized (mean age, 47.63 years [standard deviation (SD) = 9.44]; 19 [63%] males), 15 (53%) were in the CR + TAU group and 15 (73%) were in the TAU-alone group. Of these, 27 (63%), 14 (50%), and 13 (71%) in the CR + TAU, CR + TAU, and TAU-alone groups, respectively, completed the trial. The demographic characteristics were not significantly different between the groups (Table 1). Additionally, none of the groups exhibited considerable changes in medication use from baseline to 12 weeks. One patient in the CR + TAU and two in the TAU-alone groups dropped out. Reasons for dropping out of the study existed, including withdrawal of consent (*n* = 3).

### 3.2. Feasibility and Acceptability Outcomes

Overall, the results indicated retention rates of 90% and 93% in the CR + TAU and 87% in the TAU-alone groups, respectively. Furthermore, 14 patients (100%) in the CR + TAU group completed > 80% of the sessions. No accidents or injuries occurred during the CR therapy intervention.

### 3.3. Primary Efficacy Outcomes

After 12 weeks of treatment, the mean absolute change in the BACS from baseline in verbal memory was 0.75 (95% confidence interval [CI], 0.289–0.823) for the CR + TAU groups and 0.26 (95% CI, 0.251–0.808) for the TAU-alone groups; working memory was 0.92 (95% CI, 0.351–0.872) for CR + TAU and 0.03 (95% CI, 0.091–0.614) for TAU-alone; motor speed was 0.55 (95% CI, 0.572–0.982) for CR + TAU and 0.29 (95% CI, 0.386–0.909) for TAU-alone; verbal fluency was 0.57 (95% CI, 0.419–0.916) for CR + TAU and −0.16 (95% CI, 0.139–0.684) for TAU-alone; attention and processing speed was 0.43 (95% CI, 0.177–0.711) for CR + TAU and −0.11 (95% CI, 0.462–0.684) for TAU-alone; executive function was 0.62 (95% CI, 0.351–0.872) for CR + TAU and 0.06 (95% CI, 0.462–0.950) for TAU-alone; and the composite score was 0.65 (95% CI, 0.230–0.770) for CR + TAU and 0.06 (95% CI, 0.050–0.538) for TAU-alone.

**Figure 2:**
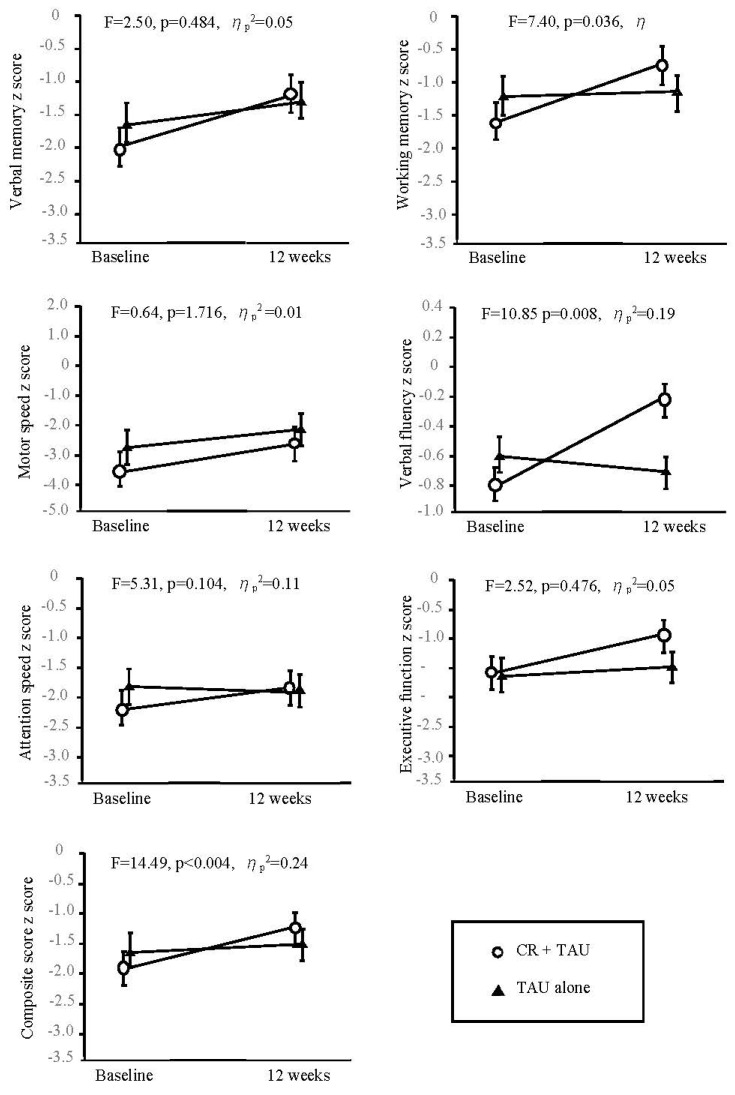
Change in BACS subtests and composite scores from the baseline to 12 weeks. The F−value in each panel is the test for the F−statistic of the interaction between time and treatment groups. Error bars indicate the standard error. BACS, Brief Assessment of Cognition in Schizophrenia; CR, cognitive remediation; TAU, treatment as usual.

The baseline assessment findings were comparable between the groups, indicating no significant differences. Table 2 and Figure 2 show the differences in the changes from baseline to 12 weeks between the groups. The differences in change from baseline to 12 weeks for working memory (F = 7.40, *p* = 0.04, ηp^2^ = 0.14), verbal fluency (F = 10.85, *p* = 0.01, ηp^2^ = 0.19), and composite score (F = 14.49, *p* < 0.00, ηp^2^ = 0.24) were significant.

### 3.4. Secondary Efficacy Outcomes

The differences in the baseline assessment results were insignificant. Table 2 shows the differences between the groups in terms of the changes from baseline to 12 weeks. The differences between groups in terms of the changes from baseline to 12 weeks were significant for QLS score (F = 47.27, *p* < 0.00, ηp^2^ = 0.50), SANS anhedonia/asociality (F = 10.66, *p* = 0.01, ηp^2^ = 0.19), and mGAF-F score (F = 18.72, *p* < 0.00, ηp^2^ = 0.29).

## 4. Discussion

### 4.1. Main Findings

This study assessed the feasibility and effectiveness of incorporating RehaCom^®^-based CR therapy into TAU on cognition and other outcomes in patients with schizophrenia at a Japanese psychiatric hospital. To the best of our knowledge, this is the first randomized trial to indicate that utilizing the Japanese version of RehaCom^®^ in CR therapy showed promising feasibility and efficacy in improving cognition in patients with schizophrenia.

This study offers encouraging evidence that adding RehaCom^®^-based CR therapy to TAU is feasible and well-received, as evidenced by high patient adherence and retention rates. Patients in the CR + TAU group successfully completed the program without worsening medical conditions and no adverse events, indicating good acceptability and tolerance. These findings suggest the feasibility of employing RehaCom^®^-based CR therapy in a Japanese psychiatric hospital. The ability to adjust difficulty levels based on the patient’s performance, a feature installed in RehaCom^®^, likely contributed to sustained motivation and engagement during CR therapy.

This study assessed the impact of adding RehaCom^®^-based CR therapy to TAU on cognition in patients with schizophrenia, finding significant improvements in several cognitive domains in the CR + TAU group compared to the TAU-alone group. These results align with that of a previous study on CR therapy using RehaCom^®^ [19,20,21] and offer robust evidence for the efficacy of CR in enhancing cognition in schizophrenia. Although the TAU group also demonstrated a trend toward improved cognitive performance, the magnitude of these improvements in the CR + TAU group was greater than that in the TAU group. To improve outcomes, implementing CR therapy alone and CR with TAU may be crucial. As McGurk et al. and Wykes et al. [12,13] reported, combining CR with other rehabilitation programs often enhances cognitive improvement compared with CR alone.

A meta-analysis by Vita et al. [40] identified “active and trained therapists”, “repeated practice of cognitive exercises”, “structured development of cognitive strategies”, and “facilitated transfer to everyday functioning” as core elements of cognitive improvement with CR. The combination of CR and TAU by RehaCom^®^ includes these four elements, and the results of this study demonstrate the importance of integrating CR and psychosocial rehabilitation. This may have had a synergistic impact on the findings of cognitive performance in the CR therapy program in this study. Targeting patients with schizophrenia may maximize cognitive benefits and improve functional outcomes. Further validation is required to assess the effects of CR-induced cognitive gains in patients with schizophrenia.

Several factors may explain the cognitive improvement found in this study. Compared to the TAU-alone group, the CR + TAU group demonstrated substantial improvement in cognition and intrinsic motivation [41,42,43], which are factors that enhance cognitive outcomes. Thus, improvements in intrinsic motivation might contribute to greater cognitive gains with CR therapy [17,41]. However, the mechanisms through which RehaCom^®^-based CR therapy enhances cognitive performance in schizophrenia are not completely understood, necessitating further validation.

The primary goal of schizophrenia treatment involves improving functional outcomes [44,45,46]. Notably, our results revealed that CR + TAU led to superior outcomes in improving the mGAF-F functional level compared to TAU alone. This can be attributed to significant improvements in cognition and intrinsic motivation. The combined benefits of CR therapy in these areas, alongside TAU, may enhance overall functional levels in patients with schizophrenia. The role of intrinsic motivation has been established [17,41,42,43,47]. Negative symptoms [44,48,49] are key elements of schizophrenia, affecting functional outcomes and mediating the link between cognition and functional outcomes. Proactive patients undergoing cumulative CR intervention can potentially improve cognition and intrinsic motivation, leading to better functional outcomes. The effects of CR interventions on negative symptoms should continue to be investigated. Furthermore, training for cognitive task sessions and verbal sessions for bridging improved cognition and real-world functioning is crucial in CR therapy [12,13,26]. The results of this study revealed that the active participation of participants in CR resulted in improvements in cognition and intrinsic motivation. One-on-one verbal sessions with an occupational therapist and psychosocial treatments included in TAU may be effective in translating these improvements into real-world functioning.

### 4.2. Strengths and Limitations

This study’s primary strength lies in the fact that we verified CR’s feasibility and efficacy using RehaCom^®^ for patients with schizophrenia for the first time in Japan. These valuable insights into the methodology of CR with RehaCom^®^ can potentially guide clinicians in implementing an evidence-based intervention to improve cognition in patients with schizophrenia.

However, this study has several limitations. Firstly, the study results were limited by a small sample size and were conducted at a single site; therefore, our findings should be considered preliminary until replicated by future studies. Secondly, the inclusion criteria may restrict the applicability of the findings to broader populations. Thirdly, since the study participants comprised inpatients and outpatients, caution is advised in interpreting functional-level assessments. Fourthly, the study did not explore the optimal dose (duration, intensity, and frequency) to maximize cognitive performance and other outcomes, necessitating further investigation. Fifthly, significant cognitive improvements observed for patients receiving CR + TAU may be the cumulative effects of computer sessions in addition to verbal sessions, not computer sessions alone; however, the design of this study did not examine the separate impacts of computer and verbal sessions on the cognition of patients in the CR + TAU group. In addition, the TAU intervention received may have varied among patients, as the TAU content was not controlled. Finally, the absence of follow-up assessments to gauge lasting effects is a limitation. Additional follow-up studies are required to determine the impact of CR interventions on cognition.

Furthermore, it has recently been reported that non-invasive brain stimulation (NIBS), such as transcranial direct current stimulation to frontal lobe regions [50,51] or the combination of NIBS and CR, may further improve cognitive function in schizophrenia [52,53]. Future studies should therefore examine the effects of adding NIBS to RehaCom^®^-based CR therapy on improving cognitive dysfunction.

## 5. Conclusions

This study demonstrates that integrating CR therapy into standard care effectively enhances cognition in patients with schizophrenia. Combining RehaCom^®^-based CR therapy with other psychosocial treatments may further elevate functional levels. These results advocate for the inclusion of this approach in schizophrenia treatment protocols. Additional research is needed to determine the prognostic significance of CR therapy.

## Figures and Tables

**Table 1:** Participant characteristics.

	CR + TAU (*n* = 14)		TAU Alone (*n* = 13)		t/χ^2^	*p*
Age (years), mean (SD)	46.57	(9.80)	48.46	(10.33)	−0.49	0.630
Sex, n (% male)	9	(64.29)	8	(61.54)	0.02	0.883
Onset age (years), mean (SD)	21.93	(6.90)	23.85	(8.38)	−0.65	0.521
Duration of an illness (days), mean (SD)	9018.00	(4115.75)	8928.46	(3540.74)	0.06	0.952
Total length of hospital stays (months), mean (SD)	111.29	(108.46)	100.69	(80.75)	0.29	0.777
Education (year), mean (SD)	12.50	(2.47)	13.15	(1.99)	−0.75	0.459
Antipsychotic						
Chlorpromazine equivalent dose (mg/day), mean (SD)						
Baseline	849.43	(603.25)	831.15	(455.17)	0.08	0.930
12 weeks	849.43	(603.25)	831.15	(455.17)	0.08	0.930
Generation, *n* (%)						
FGA	1	(7.14)	1	(7.69)	0.16	0.922
SGA	10	(71.43)	10	(76.92)		
FGA + SGA	3	(21.43)	2	(15.38)		
Prescription, *n* (%)						
Monotherapy	5	(35.71)	4	(30.77)	0.07	0.785
Polypharmacy therapy	9	(64.29)	9	(69.23)		

Student’s *t*-test for the continuous variables and χ^2^ analyses for the categorical variables were used to compare the groups. CR, cognitive remediation; FGA, first-generation antipsychotics; SD, standard deviation; SGA, second-generation antipsychotics; TAU, treatment as usual.

**Table 2:** Change in the BACS subtests and composite scores from the baseline to 12 weeks.

Outcome		CR + TAU (n = 14)		TAU Alone (*n* = 13)		*F*			Effect Size
		Mean	(SD)	Mean	(SD)	Time	Group	Time × Group	
BACS									
Verbal memory	Baseline	−2.00	(1.45)	−1.74	(1.25)	10.78 **	1.68	2.50	0.05
	Post	−1.25	(1.33)	−1.48	(1.10)				
Working memory	Baseline	−1.78	(1.20)	−1.35	(1.27)	8.29 *	4.85	7.40 *	0.14
	Post	−0.86	(1.30)	−1.32	(0.98)				
Motor speed	Baseline	−3.52	(1.63)	−2.61	(1.54)	5.28 *	0.10	0.64	0.01
	Post	−2.92	(1.51)	−2.32	(1.41)				
Verbal fluency	Baseline	−0.89	(1.16)	−0.60	(1.35)	3.52	8.51 *	10.85 **	0.19
	Post	−0.33	(1.11)	−0.76	(1.10)				
Attention	Baseline	−2.23	(1.44)	−1.81	(1.61)	1.86	4.97	5.31	0.11
	Post	−1.81	(1.47)	−1.92	(1.76)				
Executive function	Baseline	−1.62	(2.34)	−1.65	(2.23)	3.76	2.78	2.52	0.05
	Post	−1.00	(2.21)	−1.58	(2.04)				
Composite score	Baseline	−2.01	(1.17)	−1.62	(1.20)	21.51 **	11.04 **	14.49 **	0.24
	Post	−1.37	(0.99)	−1.56	(1.10)				
SCoRS									
Patient total	Baseline	35.43	(10.75)	39.08	(10.10)	20.19 **	0.07	2.10	0.04
	Post	28.07	(4.91)	35.31	(10.74)				
Patient global rating	Baseline	4.64	(2.74)	4.77	(2.42)	0.08	0.96	0.85	0.02
	Post	4.36	(2.41)	5.31	(2.39)				
Informant total	Baseline	37.43	(13.50)	38.92	(12.18)	7.68 *	3.40	2.45	0.05
	Post	30.79	(7.04)	37.10	(10.20)				
Informant global rating	Baseline	6.21	(2.05)	5.92	(1.89)	9.26 *	7.82 *	9.26 *	0.17
	Post	4.79	(1.37)	5.92	(1.98)				
Interviewer total	Baseline	38.10	(14.50)	40.54	(11.70)	11.73 **	6.56	4.76	0.09
	Post	30.79	(6.75)	38.92	(9.73)				
Interviewer global rating	Baseline	6.21	(1.93)	6.54	(1.76)	13.12 *	7.86 *	6.08	0.12
	Post	5.00	(1.30)	6.31	(1.70)				
QLS									
QLS score	Baseline	4.79	(2.49)	5.31	(2.32)	20.94 **	45.58 **	47.27 **	0.50
	Post	9.00	(3.33)	4.46	(1.98)				
IMI									
Interest/enjoyment	Baseline	30.64	(8.33)	28.15	(7.28)	1.26	9.01	6.35 *	0.12
	Post	33.86	(4.15)	26.92	(7.65)				
Value/usefulness	Baseline	35.57	(11.80)	28.46	(12.80)	1.68	4.60	2.06	0.04
	Post	40.00	(9.41)	28.23	(10.90)				
Perceived choice	Baseline	24.29	(5.76)	29.69	(5.22)	3.27	0.13	1.17	0.02
	Post	27.36	(8.18)	30.46	(4.24)				
IMI total	Baseline	90.50	(21.66)	86.31	(17.38)	5.05	7.59 *	6.51	0.12
	Post	101.36	(13.94)	85.62	(17.77)				
PANSS									
Positive	Baseline	32.00	(7.37)	32.85	(6.66)	33.22 **	1.76	2.12	0.04
	Post	26.07	(7.31)	29.31	(5.95)				
Negative	Baseline	31.00	(6.79)	32.08	(6.28)	22.86 **	2.20	2.06	0.04
	Post	26.86	(5.13)	29.85	(5.13)				
General psychopathology	Baseline	71.07	(14.37)	72.85	(12.86)	22.42 **	0.08	0.26	0.01
	Post	62.64	(11.63)	62.38	(10.14)				
Total	Baseline	134.07	(27.67)	137.77	(24.50)	39.55 **	0.33	0.18	0.00
	Post	115.50	(22.59)	121.54	(14.20)				
SANS									
Affective flattening	Baseline	29.79	(7.51)	31.85	(6.26)	22.37 **	0.97	0.53	0.01
	Post	26.21	(6.97)	29.23	(4.83)				
Alogia	Baseline	18.36	(6.03)	18.62	(5.39)	19.03 **	1.57	1.71	0.04
	Post	15.64	(4.33)	17.15	(4.93)				
Avolition/apathy	Baseline	15.29	(5.12)	17.31	(3.12)	1.37	4.07	0.36	0.01
	Post	13.86	(3.86)	16.85	(2.85)				
Anhedonia/asociality	Baseline	21.57	(4,16)	21.69	(3.84)	19.00 **	9.49 **	10.66 **	0.19
	Post	18.36	(4.80)	21.23	(3.30)				
Attention	Baseline	13.36	(3.86)	13.31	(3.88)	5.55 *	1.58	1.84	0.04
	Post	11.93	(3.85)	12.92	(3.38)				
Total	Baseline	99.64	(22.91)	102.77	(20.44)	29.95 **	5.27	5.64	0.11
	Post	86.00	(22.22)	97.38	(16.80)				
mGAF-F									
mGAF-F score	Baseline	39.14	(10.92)	41.92	(5.78)	10.77 **	14.34 **	18.72 **	0.29
	Post	49.79	(10.54)	40.46	(6.45)				

** *p* < 0.01, * *p* < 0.05. BACS, Brief Assessment of Cognition in Schizophrenia; CR, cognitive remediation; mGAF-F, modified Global Assessment of Functioning social functioning subscale; PANSS, Positive and Negative Syndrome Scale; QLS, Quality of Life Scale; SANS, Scale for the Assessment of Negative Symptoms; SCoRS, Schizophrenia Cognition Rating Scale; SD, standard deviation; TAU, treatment as usual. Estimated effect size (η_p_^2^): 0.01 (small), 0.06 (medium), 0.14 (large).

## Data Availability

This study was registered in the University Hospital Medical Information Network Clinical Trials Registry (UMIN-CTR) (UMIN000039521).

## Appendix A

**Table A1:** Classification of the RehaCom^®^ subprograms based on the MATRICS^TM^ Consensus Cognitive Battery (MCCB^TM^) classification.

Cognitive Domain	RehaCom^®^ Subprogram
Attention/Vigilance	(a) Alertness, (b) Saccadic Training, (c) Divided Attention, (d) Divided Attention 2, (e) Sustained Attention
Speed of Processing	(f) Reaction Behavior, (g) Responsiveness, (h) Vigilance 2, (i) Two-dimensional Operations, (j) Attention and Concentration, (k) Exploration
Reasoning and Problem-Solving	(l) Topological Memory, (m) Logical Reasoning, (n) Restoration Training
Working Memory	(o) Working Memory
Visual Learning and Memory	(p) Figural Memory
Verbal Learning and Memory	(q) Memory for Words, (r) Verbal Memory
Social Cognition	(s) Physiognomic Memory

This classification was based on the categories established by the MCCB^TM^.

## Appendix B

**Table A2:** Details of the treatment schedule for cognitive remediation therapy using RehaCom^®^.

	a	b	c	d	e	f	g	h	i	j	k	l	m	n	o	p	q	r	s
Session 1	○	○				○	○					○			○	○	○		○
Session 2			○	○				○	○				○		○	○		○	○
Session 3					○					○	○			○	○	○	○		○
Session 4	○	○				○	○					○			○	○		○	○
Session 5			○	○				○	○				○		○	○	○		○
Session 6					○					○	○			○	○	○		○	○
Session 7	○	○				○	○					○			○	○	○		○
Session 8			○	○				○	○				○		○	○		○	○
Session 9					○					○	○			○	○	○	○		○
Session 10	○	○				○	○					○			○	○		○	○
Session 11			○	○				○	○				○		○	○	○		○
Session 12					○					○	○			○	○	○		○	○
Session 13	○	○				○	○					○			○	○	○		○
Session 14			○	○				○	○				○		○	○		○	○
Session 15					○					○	○			○	○	○	○		○
Session 16	○	○				○	○					○			○	○		○	○
Session 17			○	○				○	○				○		○	○	○		○
Session 18					○					○	○			○	○	○		○	○
Session 19	○	○				○	○					○			○	○	○		○
Session 20			○	○				○	○				○		○	○		○	○
Session 21					○					○	○			○	○	○	○		○
Session 22	○	○				○	○					○			○	○		○	○
Session 23			○	○				○	○				○		○	○	○		○
Session 24					○					○	○			○	○	○		○	○

(a) Alertness, (b) Saccadic Training, (c) Divided Attention, (d) Divided Attention 2, (e) Sustained Attention, (f) Reaction Behavior, (g) Responsiveness, (h) Vigilance 2, (i) Two-dimensional Operations, (j) Attention and Concentration, (k) Exploration, (l) Topological Memory, (m) Logical Reasoning, (n) Restoration Training, (o) Working Memory, (p) Figural Memory, (q) Memory for Words, (r) Verbal Memory, (s) Physiognomic Memory.
